# Building and Boosting Capitals for Health Care Access: A Qualitative Study of Homeless Health Peer Advocacy in London, UK

**DOI:** 10.1111/1467-9566.70136

**Published:** 2025-12-08

**Authors:** Andy Guise, P. J. Annand, Paniz Hosseini, Spike Hudson, Sujit D. Rathod, Lucy Platt

**Affiliations:** ^1^ Population Health Sciences King's College London London UK; ^2^ Centre for Care University of Sheffield Sheffield UK; ^3^ Department of Veterinary Medicine University of Cambridge Cambridge UK; ^4^ Community Consultants Ltd London UK; ^5^ Department of Population Health London School of Hygiene and Tropical Medicine London UK; ^6^ Department of Public Health, Environments and Society London School of Hygiene and Tropical Medicine London UK

## Abstract

Peer support is widely promoted as enabling health care access with people experiencing homelessness, including through enabling independence, empowering individuals and managing stigma. However, there is little understanding of these processes, including how they relate to the wide range of potential activities involved in peer support. We studied peer support within a peer advocacy service at a charity run by and for people experiencing homelessness in London, UK. We report analysis from a sub‐set of qualitative interviews and focus groups gathered for a qualitative study of peer advocacy. We include interviews that offered insight to particular cases of peer advocacy. These interviews came from 23 clients of the service, 6 peer advocates, 1 staff member and 2 stakeholders. We drew on Bourdieu's theory of capitals to develop a typology of different ways peer support works. The findings focus on reporting three types of experiences: where peer support *builds* cultural health capital amongst service clients, *boosts* cultural health capital, social and economic capital, and third, where boosting social and economic capital are priorities. The discussion considers how the analysis helps conceptualise processes of peer support with respect to empowerment, independence and stigma management.

## 1 Introduction

Peer support is increasingly implemented in the UK and globally to support health care access with respect to experiences of homelessness (Croft et al. 2013), as well as drug use, sex work and other stigmatised identities and for a range of health issues (Chang et al. 2021; Dennis 2003; Lloyd‐Evans et al. 2014). Peer support could address the widely recognised health care access challenges faced by many people experiencing homelessness, including stigma and discrimination (Reilly et al. 2022), de‐prioritisation of health amidst interlocking emergencies (Harris 2020) and bureaucratic barriers (Elwell‐Sutton et al. 2017). Although there is growing evidence for the important role peer support can play, there is still uncertainty over the effects it can have and, importantly, how those come about. We sought to address this gap through an evaluation of a peer advocacy service run by and for people experiencing homelessness in London, UK.

### 1.1 Groundswell's Homeless Health Peer Advocacy Service in London, UK

Groundswell is a registered charity in London, UK who run the homeless health peer advocacy (HHPA) service. Peer advocates are volunteers, with some progressing to salaried posts, who implement HHPA. Peer advocates support clients who are currently homeless by accompanying them to appointments at health care services. The service provides logistical and psycho‐social support prior to appointments, facilitates communication with health care providers and staff within care interactions and supports follow‐up after appointments. Core goals of HHPA include to ‘improve confidence’ of the clients using the service, enhance clients' abilities to ‘access healthcare independently’ (Groundswell 2024) and ‘empower’ people to overcome barriers to care, including practical barriers to appointment making and registration as well as the consequences of previous negative experiences, including the stigma attached to homelessness (Finlayson et al. 2016). HHPA is increasingly recognised as enabling health care access and as potentially highly cost‐effective (Finlayson et al. 2016). There is however limited evaluative evidence for the impacts and process of how the service operates. Furthermore, claims for fostering independence, empowerment and addressing stigma need critical scrutiny given the range of possible meanings for these processes.

### 1.2 How Does Peer Support Enable Health Care Access?

Peer support has many potential forms, but involves someone with current or past experience of, for example, homelessness supporting someone who is currently homeless. Evidence from a range of fields like mental health and HIV show potential for impact of peer support (Cooper et al. 2024). Reviews have also explored the range of activities and processes that can then happen within peer support, including information sharing, mentoring and advocacy within health consultations (Chang et al. 2021; Watson 2019), while noting the ‘infancy’ of research in this area (Watson 2019) and under‐conceptualisation of processes (Chang et al. 2021). A recent review sought to respond to the limited clarity on change mechanisms in processes of peer support and homelessness (Barker et al. 2020). The review identified core elements of a working alliance between peers and their clients: clients learn behaviours modelled by peers, outcomes are mediated by peers acting as a role model and peers provide social support that adopts a range of forms. Such elements of peer support in turn potentially involve a range of components, including multiple forms of support (information, emotional and companionship), using experiential knowledge, self‐disclosure and advocacy. The review noted there is little reflection on power in how peer interventions work. Sociological literatures analysing the contexts for peer support are helpful here, by, for example, drawing attention to the challenges of managing complex overlapping identities of peer and client (Dhand 2006), how peers draw on their own social networks as a resource in peer work (Faulkner‐Gurstein 2017) and how organisational structures and policies limit peers in operationalising their role (Bonnington and Harris 2017).

The current understanding of processes of peer support is arguably defined by apparent ‘complexity’: there is understanding of how multiple potential processes can combine in different ways within a specific relationship. Although acknowledging such complexity is important, concluding that things are ‘contingent and complex’ risks not providing knowledge that can guide practice (Nettleton and Green 2014). Furthermore, claims for independence, empowerment and stigma management are challenging to conceptualise with any specificity. The apparent complexity also limits the potential for recognising and communicating impact from the service. Indeed, in early discussions with Groundswell to develop the evaluation there was reference by a member of staff to peer advocacy as ‘just talking to people’; although the emphasis on communication is important, such a sentiment—no doubt tinged with irony by the member of staff—may serve to diminish understanding of the service to outside stakeholders. This is especially pertinent when considering HHPA alongside other interventions more amenable to evaluation within currently dominant methodological approaches, that is, those reduceable to defined steps with potential for high fidelity and control across contexts.

It is in this theoretical and institutional context that we sought to develop an in‐depth understanding of peer support and specifically develop a critical understanding of claims for processes of building independence, empowerment and stigma management.

## 2 Theoretical Framework

In order to explore how HHPA operates we undertook a qualitative study integrated within a mixed‐methods participatory evaluation (Rathod et al. 2021). The overall study framing built on realist logics, in terms of seeking to understand ‘what works for who’ (Pawson and Tilley 1997) rather than a binary question of ‘does the service work’. The qualitative study also borrowed from realist logics through using concepts of outcomes, mechanisms and contexts for peer advocacy to guide the early data collection and analysis. Outcomes understood as changes, intended and unintended, resulting from peer advocacy, and mechanisms as the parts of peer work that lead to change. This framing allowed the qualitative work to integrate with other parts of the mixed‐method study, including to guide the identification of potential outcomes for a survey (See Platt et al. 2025; Rathod et al. 2021). This framing also structured initial analyses of the service we developed, to foster input from Groundswell, reflecting how logic models building on a realist approach are increasingly recognised and valued within service delivery environments.

As above, our question for the analysis here was not realist in orientation (i.e. to understand configurations of context‐mechanisms‐outcomes). Instead we wanted to critically explore the potential for empowerment, independence and stigma management and how those can be understood to come about from peer advocacy. These concepts have a common challenge of being understood either as phenomena reflecting an individual's isolated experiences and capacities or as involving power and being bound up with social context (Link and Phelan 2001; Mol 2008; Willis et al. 2016). We built on this latter tradition, especially to explore how experiences of inequality and poverty are shaped by the stigmatisation of being ‘dependent’ on welfare and state support (Peacock et al. 2014; Tyler 2020).

We developed our analysis of how peer support works, framed by Bourdieu's conceptualisation of capitals (Bourdieu 1986) and recent ideas of cultural health capital (Chang et al. 2016; Shim 2010). Capitals here are the resources an individual accumulates through life, and so represent inequalities in society. For Bourdieu, these capitals are principally social, economic and cultural: social capital denoting durable networks of relationships; economic capital being resources convertible to money; and cultural capital as competence with dominant forms of culture, for example, modes of language, arts or music (Bourdieu 1986).

Economic and social capital have received considerable research attention with respect to health, although there has been less attention to cultural capital (Abel 2008; Pinxten and Lievens 2014). Shim responded to this gap through developing the concept of cultural health capital to refer to ‘verbal and nonverbal competencies and interactional styles that can influence health care interactions at a given historical moment’ (Shim 2010). Cultural health capital is a resource that can be ‘exchanged’ to allow the success of health care interactions. Examples of such capital include patients having the skill to deploy or follow particular language recognised as legitimate by health care providers, to signal potential to hold providers to account or to control and manage stigma or emotions in a care setting (Chang et al. 2015; Dubbin et al. 2013; Gengler 2014; Rasmussen et al. 2021).

We used the framework of capitals in our analysis to interpret what peers and clients described to us. The idea of transmitting or transferring cultural health capital or other capitals provides a way to conceptualise empowerment and independence. For Bourdieu, cultural capital is developed slowly over time, subject to the efforts of the individual agent (1986). Other studies have suggested that cultural health capital ‘develops’ through constant presence in health care settings and questioning providers (this in the context of parents and their ill‐children; Gengler 2014) and that people can develop this cultural health capital over time and across multiple health care encounters (Willis et al. 2016). In another analysis on the impact of HHPA on peers themselves we noted that peers' knowledge and experience may operate as cultural capital (Annand et al. 2022). However, existing literature to date has not considered in‐depth how peer support relates to cultural health capital in health care access. Experiences comparable to peer support have though explored the role of lay and community health workers and social capital. Studies suggest that different capitals are ‘exchanged’ by workers and thereby increase access to capitals for particular individuals or communities (Adams 2020; Saint Onge and Brooks 2021). Group health care visits facilitated by clinicians have also been described as sites where social and cultural health capital are cultivated (Thompson‐Lastad et al. 2025). Through this analysis we sought to build on this literature on capitals and especially to think about how capitals are ‘transferred’, ‘cultivated’ and ‘exchanged’.

## 3 Methods

Our design combined different sources of qualitative interview data collected iteratively in the context of immersing ourselves within the ongoing operations of HHPA. Although informed by ethnographic approaches, we were pragmatic in line with the demands of health services research and the emergence of COVID‐19 in March 2020.

We had an ongoing dialogue with Groundswell through the study and sought to embed ourselves within the organisation. We had a regular presence in Groundswell's offices and joined in team meetings and training sessions. This was not for formal data collection but to assist in our understanding of the context for HHPA and to identify avenues for the ongoing research. People with lived experience of homelessness were also part of the research team, collecting data and assisting in the analysis and interpretation of the data.

We collected data through individual and group interviews with HHPA clients (*n* = 29), non‐clients (*n* = 10), peer advocates (*n* = 13), Groundswell staff (*n* = 2) and stakeholders (*n* = 15) working in health and homelessness services across London. These different data sources were combined to both triangulate and seek breadth of insight. For example, accounts from clients and peer advocates were triangulated to explore how peer advocacy operated.

We mainly used purposive sampling with a goal of generating information‐rich accounts. With respect to clients of the HHPA service, we sought a range of respondents by race and ethnicity and then gender. Strategies for recruitment varied. HHPA clients were recruited either through Groundswell or indirectly, whether through hostel staff or through clients completing the survey from the cohort component of the mixed‐method evaluation and giving permission to be contacted for an in‐depth interview. From stakeholders we included different positions in the health and homelessness system, such as health care professionals as well as outreach workers, hostel staff and service commissioners. Sampling was also driven by convenience, with peer advocates we interviewed being anyone from the Groundswell team who was willing to talk to us, reflecting a limited pool of interviewees to draw from.

Interviews in the opening months of data collection—from August 2019 onwards—were exploratory and asked respondents to describe their experience of peer advocacy, with a particular goal of defining core parameters of the service to inform the cohort study within the wider evaluation. Later we refined an interview schedule that sought to explore different aspects of HHPA, asked for reflections on Groundswell and peer advocacy and then finally what impacts they considered the process to have. Reflecting the multiple contingencies of the interviews we were not always able to cover all areas. For example, exploring with clients what exactly they had received in terms of HHPA and when could be difficult, reflecting recall challenges of what are often routine processes (as for any research on health care use across populations). Linear narratives of how a service had first been used and then evolved through each subsequent appointment were rare. Instead, and we encouraged it, respondents' accounts were structured by reference to their own needs and perspectives. Respondents were also often managing trauma that led us to be cautious in exploring experiences in‐depth, and instead led us to follow respondents in what they wanted to share.

COVID‐19 had significant impact on the study, with data collection stopping during the first UK lockdown from March 2020. During this time, we stayed in close contact with the Groundswell team whilst they focused on reorienting the service. We then began remote telephone interviews from Autumn 2020 until mid‐2021 and continued with a small number of interviews in the summer of 2022. Although many people experiencing homelessness in the UK either have phones, or have access to phones through hostel staff, this nonetheless posed limits on who we could talk to. For example, it limited our research to people who are in homeless hostels, rather than also including experiences of rough sleeping, although Groundswell tend to work more with people in hostels. Similarly, we had planned to observe peer advocacy, follow up on interviewees to understand change over time and include people who did not speak English (some interviewees had English as a second language). Social distancing measures made observation impossible for an extended period. Across all of these plans we faced limits on logistics and resources: arranging an interview over the phone was very challenging, and doing this across other languages with an interpreter even more so. Research staff time also became limited, with project resources being taken up in the extensive and ongoing reorganisation of the study.

The COVID‐19 pandemic also brought adaptations to HHPA: welfare phone calls were introduced to maintain contact with clients and offer support, as was the widespread use of private taxis to get people to appointments, sometimes without a peer advocate there. Such innovations raised the question: what were we evaluating? Were welfare calls by peer advocates part of HHPA? Was a taxi to take someone to an appointment without a peer being there HHPA? Our approach, here, is to focus on understanding peer advocacy as a process of supporting people to attend health care appointments, and to consider welfare calls and arranging taxis as they relate to that process. Considering these components helps explore the boundaries of what peer advocacy is and how it operates.

Our analysis followed iterative coding strategies following principles of abduction and seeking to work with and build on existing theory (Corbin and Strauss 2008; Green and Thorogood 2014; Tavory and Timmermans 2014). As data were collected we familiarised ourselves with it, and implemented initial coding and memo writing; tentative hypotheses then fed into ongoing data collection. This early analysis also fed into an initial coding framework, analytical thoughts and ‘logic models’. As described above, we used realist concepts of contexts, mechanisms and outcomes to organise our emerging codes. We drew on the review by Barker et al., cited above, to develop some codes that described how peer support works, adapting these in line with our data; this included, for example, drawing on concepts of role modelling and different forms of support, including information, resource and emotional support. At other times core codes emerged from the specifics of the data, for example, the concept of ‘fighting your corner’. After data collection finished, we discussed and finalised a coding framework, principally amongst A.G., P.A. and P.H.; second, coding and team discussion were used to explore codes across a selection of the data. Final coding and analysis of the dataset was conducted by one member of the team, A.G..

As coding progressed we used the capitals framework to develop a typology of how HHPA works. We looked across the dataset to identify how cultural, social and economic capitals were transmitted or exchanged. The analysis presented uses the different capitals to group together experiences of peer advocacy described to us. The analysis reported below draws from a sub‐sample of our overall interview dataset. Although all interviews explored peer advocacy from different perspectives and described elements of it in‐depth, not all interviews offered detailed narratives of how clients used peer advocacy and what outcomes or patterns of ongoing use it led to. In the analysis here we focus in on these ‘cases’ of peer advocacy. Within each ‘case’ of an experience of peer advocacy described to us—whether accounts from individual clients or peers, staff or stakeholders describing clients' experiences—AG looked across the various factors coded for and interpreted a ‘line of argument’ that placed the account within an emerging typology of capitals. Crucially, the accounts the analysis is based on vary: for some ‘cases’ there were clear narratives that link together mechanisms and outcomes with particular aspects of HHPA, at other times possible links between different mechanisms or outcomes are inferred or only mechanisms or outcomes are clearly articulated. In both situations the analysis is limited by being based on accounts of care and any change following it, which offer important but necessarily limited insight into what happened and what processes of change there might have been. We return to this in the limitations section. The emerging analysis was regularly discussed across the research team and with Groundswell peer advocates and staff.

### 3.1 Ethics

The study had ethical approval in the UK from NHS Dulwich Research Ethics Committee (IRAS project ID 271312) and King's College London (ref HR‐18/19–11,523). Individual participants are anonymised to protect confidentiality. Groundswell are named in the paper owing to the research funding that publicly described the programme being evaluated.

## 4 Results

As described in the methods, the specific analysis reported here to develop a typology of capitals only draws on interviews where a case of peer advocacy was described in some detail; the data reported here then primarily draw on interviews with 23 clients of the service, 6 peer advocates, 1 staff member and 2 stakeholders. The clients interviewed had a range of experiences and identities. Some had been homeless for extended periods of time, involving rough sleeping, squatting and hostels, whereas others had been temporarily housed by the council and were now housed. For all the clients interviewed, respondents ages ranged from 23 to 64 years old; 7 identified as women, 1 as non‐binary, and the rest as men; 3 described themselves as bisexual, 1 as gay, 1 queer and 21 as straight. 14 identified as White‐British, 3 as mixed race (British and Black–White and Arab–White, and then European White–Black), 1 as British–Asian, 3 as Black‐British, 2 as eastern European, 1 as British–African, 1 as British–Caribbean, and 1 as Nigerian. Past or current use of drugs and alcohol was reported by 15 respondents. Twenty respondents described themselves as disabled. Health histories were varied, with narratives often addressing long series of illness, injury and disease, whether cancer, liver disease, depression, anxiety, injuries, stroke, Hepatitis C or dental damage. As above, the contingencies of the interviews meant we did not collect some information from some respondents. We withhold summary of peer advocates owing to potential risk of indirect identification within a small organisation. In the analysis below we contextualise individual health and life experience when interpreting the data.

### 4.1 Building and Boosting Capitals

As became apparent through the early stages of the study, HHPA works in a range of ways and did not have obvious focus on particular health outcomes. Instead, accounts from peer advocates, staff and clients commonly emphasised or revealed flexibility in the work done and how the emphasis was on responding to what the client wanted, with an explicit or implicit offer of open‐ended support: ‘we are here as long as you need us’ being one motto reported to us. Following this we explore three main ways in which HHPA works to *build* or *boost* specific capitals. By *building* capitals we mean how those using the service experience an enduring, perhaps permanent, change, specifically in cultural health capital. Here, capitals are ‘transmitted’ by peers to clients (Bourdieu 1986). By *boosting* capitals we mean a more temporary phenomena, bound to the particular episode of care, where clients' capitals are increased in ways entirely contingent on the presence and work of the peer advocates. Boosting capitals could involve, as we describe, clients borrowing capitals, having peers exchange their capitals on clients' behalf or transferred to them in ways that do not endure. Using this framework we describe three types of experiences: (1) cultural health capital being built, (2) cultural health capital being boosted, as well as social and/or economic capitals or (3) where cultural health capital is not needed and social and/or economic capitals are boosted. As we note, these types are not discrete categories, with experiences crossing the types at different points.

### 4.2 Building Cultural Health Capital

Some accounts described what we frame as clients' cultural health capital being built. Here clients' skills and competencies to influence health care are enhanced which is narrated as leading to people accessing care independently. We explore this idea of building cultural health capital for how it corresponds to a ‘classic’ narrative of peer support empowering people and growing independence in care. This extract from Peer Advocate 3 is one example:

my way of like working with clients is … for them to start feeling that they have a right to this interaction to these different things, and there’s a degree of coaching. Also there’s a lot to do with how one actually interacts with GPs and consultants and stuff like that.… I might take them to their first hep C treatment and, you know, and then, but like because essentially things have just gone really well, like they’re basically okay and going on their own to them. They might ask for help with [bus or train] fares and stuff like that and I can arrange that….once they’ve basically, you know, like they’ve got the routine going with that, they can get there by themselves and they build up a relationship with the people doing the dressings and so they can do that on their own. … so yeah, like that’s kind of what can, you know, it’s just people’s confidence sort of comes back once they’ve actually started to re‐engage with like services.

A central process referenced by Peer Advocate 3 is people being coached on how to interact with health care professionals. Growth in those specific competencies—cultural capital—is in turn bound up with growing ‘confidence’ in care, and people feeling they have a ‘right’ to be there. The economic capital of support for transport costs is mentioned, but not primary to the account.

A sense of a slight growth in cultural health capital also comes from Client 2 who described the peer advocate making her ‘feel that I could do it’ (i.e. go to care). A peer advocate joined for the first of her Hepatitis C treatment appointments, and then Client 2 carried on afterwards by themselves. The peer not only helped keep her calm when she was panicking but also ‘explained the way things was going to happen…so I knew what I was going into’. There are then steps of informing people on the intricacies of health care systems which can work to build cultural health capital that foster a sense of peoples' own capacity to manage care.

That accounts of building cultural health capital were not common within the dataset we attribute partly to limits on sampling: given the context of COVID‐19 especially, it was hard to find people who were previously in contact with Groundswell but now no longer, because they were now independently accessing care (and so potentially because their capitals had been built, as distinct from the processes described below). The method of relying on accounts of care at a single point in time is also limited: what we describe below as a ‘boost’ to cultural health capital below could possibly, overtime, build capital. However, many more of the accounts in our data corresponded to the idea of boosting cultural health capital.

### 4.3 Boosting Cultural Health Capital and Social and Economic Capital

We derived the notion of a ‘boost’ to capitals from respondents' accounts of the process. Peer 2, in describing their role in articulating client need says: ‘I can talk to a doctor and tell the doctor what to do, like a boost, like it's a boost, you know?’ Crucially, such a boost in capitals—in this instance providing the cultural health capital to make an interaction work—might only have impact within a particular care episode. Such a boost may not be needed or wanted every time care is needed, nor does it figure necessarily within a process of clients' capitals being built. This idea of boosting capitals for the care episode is significant for complicating ideas of how peer advocacy is supporting independence and empowerment. Here we explore how cultural health capital—particular competencies and skills to influence health care—is provided by the work of peer advocates in support of what clients needed.

Client 10's experience is indicative here. They had been using the HHPA service for over 6 months, for an ‘uncountable’ number of appointments in their words. They had experienced or were managing multiple health issues, including injuries, liver disease, mental health problems and other conditions, and at the time of the interview were in a hostel. They described how they struggled with paperwork and would ‘put their head in the sand’ when it came to organising health care, and this in the context of feeling ‘dismissed’ by health care professionals in the past. A core part of this account was of peer advocacy getting the bureaucracy to work including through complaining on their behalf. As they described:

Client 10 they make some people accountable when they're not dealing with you correct. …

Int …Can you give me an example of that, where they've made somebody accountable?

Client 10 Well, basically, so for [clinic], like I'd been waiting for like, maybe like a month. So they got me appointment and then I had a worker. Then she just went missing. HHPA Phoned up her boss, said, ‘What is this lady doing?’, you know what I'm saying?

Int Yeah.

Client 10 Like she's not supporting her client, things like that. Phoned up the hospital like, yeah, you was meant to give him an appointment, he's still waiting. Like what's going on, like, just things like this.

Client 10 here we see as being boosted, with Groundswell using their cultural health capital of knowing how the health system works, to take action on the client's behalf, in supplement to what the Client already has, and the action already taken. In addition, core to the process is a peer giving emotional and moral support, and being present and available throughout. Client 10 is attending care, and getting necessary health support, even if not in ways that fit comfortably with a narrative of ‘empowerment’ to achieve ‘independence’. Client 10 adds about their experience: ‘if I would have knew this was what you could get, I would have reached out long ago’. Client 10 is not without agency: a level of cultural health capital is needed to recognise the support others can give and seek it. Here the idea of peers providing a short‐term boost to capitals is helpful: there is not always an enduring change in the capitals clients have access to, and so the role for peers might be ongoing. For Client 10, however, permanent change might be a feature in the future: they were relatively recently out of prison, following a life history characterised by violence and hardship and now managing multiple physical and mental health problems. At this period Client 10 needed a ‘boost’, but in future they might also build cultural health capital, as indicated in expressing a wish to be a peer advocate themselves in future.

For other clients the boost was to cultural, social and economic capital. Client 16 was managing multiple health issues, combined with disability and not being able to read. He describes the peers as ‘guardian angels’, helping with a range of social needs, and then also providing social support and especially in getting the health system bureaucracy to work. The combined effect of this work has then fostered hope and confidence, as client 16 says:

them people, they’re in my heart, they’re my best friends, you know what I mean, like they brought, they brought… They brought life back, they brought… You know, I’m grateful, I’m grateful, that’s all I can say, I’m grateful.

### 4.4 Boosting Social and Economic Capital

For other clients there was more emphasis on boosting social and economic capital; these accounts were the most common in our data. Here any action is not necessarily leading to lasting change in social and economic capital, but instead primarily works for the duration of a particular appointment.

This mode of advocacy is notable for how there is, in a specific sense, potentially little ‘advocacy’ done. Processes here were often instead oriented to addressing logistical and transport issues or taking other action outside the consultation room. For Client 22 they described the process in simple terms:

they [peers] just come, knock the door, they knock the door, we went to the appointment and they sit down and wait for whatever you’ve got to do, whatever you’ve got to do with the doctors, whatever and then bring you home, that was it, that was all I was doing, I don’t know if they do anything else.

Other accounts were similar in briefly emphasising a role for peer advocacy in arranging transport and logistics, extending to a peer not even being present when, for some clients, they requested just a taxi to get them to care. Client 27, for example, was adamant that they just wanted the taxi for their regular dialysis appointments, declining Groundswell's offer to come in and support them in the hospital appointments. But other accounts indicated more. Client 22's brief portrayal of the process at the start of the interview evolves to valuing the social support in the context of being blind:

all the taxi men are doing is driving you to the building, walking you into reception and leaving you and then will pick you up… when you sit in the waiting room you’re thinking gee, I can’t even pick up a magazine, I can’t do nothing, I can’t even, you’ve got a picture on the wall, I can’t see anything…That’s what I’m saying, so when go with Groundswell, at least I’ve got somebody to talk to.

Brief accounts from clients focussing on addressing particular care barriers might then have multiple meanings. In some situations, indicating people only needed and wanted transport and logistical support. At other times they could indicate a reluctance to seek some forms of support. C15 emphasises through brief responses to questions in the interview that the peer is there to help him get around with his wheelchair, although he also references having a coffee several times, suggesting its importance. Later on he says the peer is there and ‘helps with the taxi, yeah, basically to be as independent as possible’ and later if asked if he would like to see the peer more or less he says:

Client 15 when I need him, when it's needed, I mean that's, you don't understand, I don't like to ask for things, and…

Int Yeah, sure.

Client 15 …I do what I can, if I can't do it I have to rely.

The desirability of independence figures in the account, and perhaps limiting potential for the interview to explore other things that might suggest ‘dependence’, in the same way they were reluctant to talk about the extent of their multiple health problems, as they say ‘I'm in a shit situation, but its better than a tent’. Here our data suggest that although some clients mainly sought a boost to economic and social capital there may also be scope for more support. Some peers and Groundswell staff accounts support this by suggesting that an initial appointment for something like registering at the dentists and the opticians could over time evolve into a deeper understanding of need and so continued work on other issues.

Client 29 offers insight to how experiences cross the boundaries of our typology. At times they just used a taxi to get to appointments without a peer advocate, but at other times sought help in managing the bureaucracy although this part of their account was less clear. Therefore, here Client 29 is at times wanting a boost to economic capital, and at other times potentially wanting a boost to cultural health capital, and this process of seeking out distinct support is something they explained:

I'm a very independent person. If you were to see me to walk down the road and if we weren't going to have this conversation, and just have a normal conversation, you wouldn't think anything was wrong with me. You wouldn't think I've been homeless. You wouldn't think I've had any problems. You know anything about my physical health, let alone mental health unless I'm going mad at someone. But like, you really wouldn't. And so, like I fall in the independent, very independent to the, I actually need a lot of fucking help, category.

## 5 Discussion

We sought to explore how a homeless health peer advocacy service in London, UK works, including assessing claims for empowerment, independence and stigma management. Our analysis described processes of building or boosting cultural health capital, and processes where cultural health capital is not needed; across these, different configurations of economic and social capital were priorities.

The analysis offers a route to engage with the complexity of peer support and discourses of empowerment and independence. Here, access to cultural health capital, as well as social and economic capital, and how they can be in different ways ‘built’ or ‘boosted’ point to different experiences of empowerment and potential for independence in accessing care. In particular, the analysis describes experiences of peer support that hitherto have not been the focus for policy and programme debate, for how they do not fit conventional notions of independence and empowerment. The three themes presented highlight a range of experiences of peer support without either simplifying (‘its just talking to people’) or resorting to claims of ‘complexity’, and in so doing can foster greater recognition of the possible impacts that peer support has.

The first theme of building cultural health capital we see as representing a ‘classic’ front stage account of peer advocacy: through peer support an individual's capacity to access health care changes, and they are then accessing care alone afterwards. Our analysis points to how this is happening and that a process of empowerment—cultural health capital being built—is likely a process of small and incremental change, even if the consequences for a particular individual are substantial.

The process of boosting capitals, both cultural health capital and social and economic capitals, helps to critically develop the notion of how peer support works by highlighting processes that are either undervalued or harder to talk about within a particular funding and cultural environment. Cultural heath capital, and other capitals, being boosted is not a ‘classic’ pathway towards ‘independence’. Instead the approach here could imply ongoing forms of use of the service, falling within the bounds of what is often stigmatised as ‘dependency’ (Peacock et al. 2014). Such potential stigma we see as explaining why this process figures less in the ‘front stage’ accounts of peer support. Our interpretation of this process though builds on critiques of claims for independence: whether independence, in health care and beyond, for *anybody* is ever possible or desirable (Sennett 2003) and more specifically of how such ideas represent historically and socially specific imaginings of a privileged few (Mol 2008). Following this, we see such ‘dependence’, in the form of having capitals boosted, as an important process for health and welfare: necessary care appointments are attended, fears of stigmatisation are addressed, ill‐health is attended to, and this occurs in contexts of often long‐running social and economic exclusion. Being ‘boosted’ is likely the optimal outcome of the process in the context of, for example, ongoing cancer care or disability. Further, if we put aside notions of ‘building independence’ such co‐produced processes between peer and client are understandable as empowering: through HHPA people's access to social, economic and cultural resources is being enhanced to complement their existing capitals. Here we challenge ideas of individual empowerment, and relate it to conceptions of ‘power with’ rather than just ‘power to’. People might not be entirely independent—if such a phenomenon is ever possible—but with others they are enabled to achieve health related goals.

The experience of boosting social and economic capital but not cultural health capital, as we described to be the case for some clients, raises additional questions as to what such ‘dependence’ on peer support may be for. In such situations it is probable that for any single health care appointment a client might have gone anyway even without a peer advocate being there. From the perspective of an individual appointment, peer support might then be seen as unnecessary, or at least an ‘inefficient’ use of resources (though given the voluntary nature of the HHPA service, costs are relatively low). Based on our analysis we anticipate that peer support may make a fundamental difference over the long‐term, when considering multiple appointments and processes in combination. For example, the social or symbolic role of a peer advocate can make difficult processes more bearable: preventing the effects of stigmatisation, or countering the fear of an upcoming (and potentially painful or upsetting) clinical intervention whilst waiting in a busy and uncomfortable waiting room. By making care more bearable there is greater potential for long‐term care engagement. The importance of fun, sociability and unbounded support also points to other goals for health care, beyond ‘good health’ that may not be achievable. Goals might instead be maintaining welfare and dignity in the context of chronic illness, cancer, trauma or violence. Here, an ongoing boost to economic, social and cultural capital might be essential for welfare.

A final consideration is how the building or boosting of cultural health capital, or boosting of social and economic capital may confer symbolic capital: the resource of being considered as important for others and, therefore, for oneself (Bourdieu 2000, 240–242). Symbolic capital is not a form of capital on its own, but instead other capitals function as symbolic capital in specific social interactions: namely, when holding specific capitals in relation to agents predisposed to recognise—or more accurately, misrecognise—those as important. Possessing a negative symbolic capital then equates to being stigmatised (Bourdieu 2000). The notion of being considered important, and so considering oneself important, relates to the multi‐dimensional and highly situated nature of stigma: of stigma variously being potentially gradually internalised, anticipated in particular settings and bound up in both discriminatory actions and the creation and maintenance of inequalities and status hierarchies (Link and Phelan 2001; Parker and Aggleton 2003). Our results suggest specific ways in which symbolic capital could be conferred, and so by implication stigma could be being managed. For example, how developing cultural health capital can allow potentially discriminatory interactions to be avoided, or of how a boost to social capital or economic capital is a marker of others—whether Groundswell, or a health system—valuing you. Equally, other data suggest that boosting some capitals—perhaps social capital—whilst signalling importance, also risks a negative symbolic capital in some interactions through drawing attention to stigmatised experiences of being dependent on others. Our analysis is limited here, but this brief discussion points to the highly specific social interactions in which stigma might be managed through peer advocacy. Further, it suggests directions by which Bourdieu's theory of symbolic power could be further applied to analysis of stigma (Guise 2024; Parker and Aggleton 2003).

The analysis here contributes to the existing literature on capitals for health care access from Shim (2010) and Chang et al. (2016) and especially the role for comparable processes to peer support in fostering these (Adams 2020; Saint Onge and Brooks 2021; Thompson‐Lastad et al. 2025). The notion of boosting capitals as well as building them adds to existing notions of capitals being transferred, exchanged or cultivated. Our analysis also adds to this body of theory by suggesting peer support enables health care access through combinations of capitals. Other studies have suggested different ‘clusters’ of economic, cultural and social capital relate to different experiences of health care uptake and use, with all three contributing, but different capitals contributing to different forms of care (Paccoud et al. 2020). Through suggesting specific ways in which capitals cluster and can be supported, our analysis helps develop understanding of how health services can respond to long‐running social inequalities. This is the core contribution the capitals framework we used to interpret our data could have: this framework offers potential to understand peer support with reference to social need grounded in life trajectories and position in society and social hierarchies, rather than emphasising disease outcomes or progression in health status. Future research could further explore these clusters of capitals by examining how they relate to specific intersections of discrimination and identity in particular social settings, and especially do this through close consideration of how particular clusters of capitals confer symbolic capital, or not, in particular settings.

Our analysis was limited in its engagement with the historical experiences of the clients we interviewed, and so in understanding more fully the capitals available and how, following Bourdieu, access to capitals is determined by social position; furthermore, our purposive sample was diverse in important respects, but the data collected and analysis did not generate specific insight into the role of any intersecting axes of identity. Future work could then explore whether the necessity for building or boosting capitals—cultural, social and economic—can be related to particular social and systemic contexts and the experiences of exclusion and stigma within them. For example, are needs associated with Hepatitis C treatment in the context of drug‐related stigma more amenable to having cultural health capital built? On the other hand, might health system racism in the context of homelessness necessitate, for example, more focus on boosting social and symbolic capitals within care encounters? We noted above that there were limits on exploring the experiences of people currently rough sleeping, as distinct from those staying in hostels or temporary accommodation; on the basis of our analysis here, we suggest that boosting symbolic capital may be a priority—beyond cultural, social and economic capital—in the context of such experiences. Our analysis primarily focused on cultural, economic and social capital, responding to existing themes in the literature and a close reading of our data. We acknowledge though that Bourdieu's theory arguably emphasises symbolic power and capitals, and that different readings of the analysis here and future work could place primacy on symbolic capital as a focus for enabling health care access and especially in how that functions to manage stigma.

The principal implication of the study is to provide a framework by which the complexity and range of experiences of peer support can be explored within Groundswell and policy and evaluation debates in the UK and other settings. As described, current policy and evaluation approaches risk negating much of the work that peer support achieves, either through simplistic understandings of what achieving independence or empowerment are, or by downplaying the full consequences that ‘talking to people’ can have. The framework could be a basis for developing novel indicators of programme outputs and success that more closely resemble the full range of experiences of peer support. Further, the typology we have described, and continuing work in this area, could be useful for Groundswell and other agencies in planning and training for the work of peer support. This analysis could also help with targeting support at particular clients and anticipating particular ‘pathways’ of care. We do recognise that such a suggestion is in tension with the benefits of a purposefully flexible service, and that understanding need is often in itself a long‐term process that unfolds over time. The framework could, however, at least support training efforts for future peer advocates, by giving new ways of communicating the complexity and variety of the work. The findings should also prompt all stakeholders, especially those involved in funding and planning services, to recognise that goals for empowerment and independence in care need to be understood more expansively, and especially to recognise that ongoing and open‐ended use of peer support services is potentially integral to good health and realising specific health care goals.

## 6 Limitations

We have already noted the constraints of COVID‐19, and how that led to changes in the health service and Groundswell, as well as limited data collection, especially with respect to understanding in‐depth experiences of people who do not speak English and who are currently rough‐sleeping. The research design that focused on single interviews to generate accounts of care can give some insight into potential processes of care and change in response to them. However, the analysis here is necessarily limited, in that such accounts can only give partial insight into what we hoped to understand. Future research should further develop the typology of capitals described by seeking to foster more triangulation across data sets, especially combining analyses such as that described here with repeat interviews, ongoing observations of care interactions and other social contexts and assessment of health records and other quantitative indicators indicating change in health access and status.

## 7 Conclusions

Through analysis of the homeless health peer advocacy service run by Groundswell in London, UK we conclude that this form of peer support enables health care access through building or boosting cultural health capital, as well as boosting social and economic capital. Future service developments and research should explore how this framework can support targeting of training and support as well as inform detailed monitoring and evaluation.

## Author Contributions

**Andy Guise:** funding acquisition, supervision, conceptualization, data curation, methodology, project administration, investigation, formal analysis, writing – original draft, writing – review and editing. **P. J. Annand:** conceptualization, data curation, methodology, project administration, investigation, formal analysis, writing – review and editing. **Paniz Hosseini:** investigation, formal analysis, writing – review and editing. **Spike Hudson:** investigation, writing – review and editing. **Sujit D. Rathod:** conceptualisation, writing – review and editing. **Lucy Platt:** funding acquisition, supervision, conceptualization, writing – review and editing.

## Funding

The project was funded by the National Institutes for Health Research (ref PHSEZO60).

## Ethics Statement

The study had ethical approval in the UK from NHS Dulwich Research Ethics Committee (IRAS project ID 271312) and King's College London (ref HR‐18/19–11,523).

## Consent

All participants gave informed consent.

## Conflicts of Interest

The authors declare no conflicts of interest.

## Permission to Reproduce Material From Other Sources

The authors have nothing to report.

## Data Availability

Research data are not shared.
